# Combined transcriptomic and metabolomic analyses elucidate key salt-responsive biomarkers to regulate salt tolerance in cotton

**DOI:** 10.1186/s12870-023-04258-z

**Published:** 2023-05-10

**Authors:** Mingge Han, Ruifeng Cui, Delong Wang, Hui Huang, Cun Rui, Waqar Afzal Malik, Jing Wang, Hong Zhang, Nan Xu, Xiaoyu Liu, Yuqian Lei, Tiantian Jiang, Liangqing Sun, Kesong Ni, Yapeng Fan, Yuexin Zhang, Junjuan Wang, Xiugui Chen, Xuke Lu, Zujun Yin, Shuai Wang, Lixue Guo, Lanjie Zhao, Chao Chen, Wuwei Ye

**Affiliations:** 1 Institute of Cotton Research of Chinese Academy of Agricultural Sciences / Zhengzhou Research Base, State Key Laboratory of Cotton Biology, School of Agricultural Sciences, Zhengzhou University, Anyang, 455000 Henan China; 2 Anyang Institute of Technology, Anyang, 455000 Henan China

**Keywords:** Metabolome, Transcriptome, NaCl, Mechanism

## Abstract

**Background:**

Cotton is an important industrial crop and a pioneer crop for saline-alkali land restoration. However, the molecular mechanism underlying the cotton response to salt is not completely understood.

**Methods:**

Here, we used metabolome data and transcriptome data to analyze the salt tolerance regulatory network of cotton and metabolic biomarkers.

**Results:**

In this study, cotton was stressed at 400 m M NaCl for 0 h, 3 h, 24 h and 48 h. NaCl interfered with cotton gene expression, altered metabolite contents and affected plant growth. Metabolome analysis showed that NaCl stress increased the contents of amino acids, sugars and ABA, decreased the amount of vitamin and terpenoids. K-means cluster analysis of differentially expressed genes showed that the continuously up-regulated genes were mainly enriched in metabolic pathways such as flavonoid biosynthesis and amino acid biosynthesis.

**Conclusion:**

The four metabolites of cysteine (Cys), ABA(Abscisic acid), turanose, and isopentenyladenine-7-N-glucoside (IP7G) were consistently up-regulated under salt stress, which may indicate that they are potential candidates for cotton under salt stress biomarkers. Combined transcriptome and metabolome analysis revealed accumulation of cysteine, ABA, isopentenyladenine-7-N-glucoside and turanose were important for salt tolerance in cotton mechanism. These results will provide some metabolic insights and key metabolite biomarkers for salt stress tolerance, which may help to understanding of the metabolite response to salt stress in cotton and develop a foundation for cotton to grow better in saline soil.

**Supplementary Information:**

The online version contains supplementary material available at 10.1186/s12870-023-04258-z

## Background

The effects of global warming have increasingly led to devastating environmental stresses, such as heat, salinity, and drought. Soil salinization is one of the most harmful abiotic stresses, affecting 7% of the world’s land area and 33% of irrigated lands worldwide [1]. The proportion of arable land facing salinity is expected to rise due to intensified human-driven climate change, exacerbating the threat to global food security for the exponentially growing populace. Agricultural losses from saline soils were estimated to be as high as $27.3 billion per year, according to the Agriculture Department [2]. Currently, research reports estimate that 33% of irrigated farmland and 20% of arable land were highly salinized, with an expected annual increase of 10% [3, 4]. The excessive use of chemical fertilizers, improper irrigation, industrial pollution, gradual depletion of the ocean and the weathering of minerals have exacerbated the salinity of these soils. Some fertilizers have high salt content and low water-holding capacity, and after evaporation, the salt concentration in the soil is higher [5, 6]. Therefore, in the foreseeable future, soil salinization will still be a huge environmental problem. It is estimated that 50% of arable land will be salinized by 2050 [7]. NaCl is the most important salt in nature and the main component that causes salt stress. Crops produced in soil affected by salinity exhibit a complex series of physiological, morphological and biochemical processes, resulting in very low crop yield and quality, and even toxicity [8]. In addition, soil salinization not only affects crop yield but also affects the physiological and chemical properties and ecological balance of the affected areas [9].

Under salt stress, plants will adjust in time at the molecular, cellular, protein and metabolite levels to adapt to the survival of salt stress. The production of metabolites is the final result of the plant's defense against the external environment, and it tells us what has happened in the plant when it is under stress. This metabolomics is considered a powerful tool to study in detail complex metabolite changes associated with salinity responses [10]. This understanding of plant metabolome changes under salinity could provide clues for improving the salt tolerance of affected plants to optimize crop productivity and quality in stressed plants [11]. At the same time, plant metabolomics contributes to agrobiodiversity screening, which is a potential tool to study plant stress resistance mechanisms and to develop biomarkers for stress resistance breeding. Investigating metabolomic knowledge and gaining a comprehensive understanding of the complex mechanisms that control plant salt tolerance are essential for improving crop yields and food security. Therefore, using metabolomic methods to analyze the changes in metabolites to study the metabolic regulation of plants under stress has attracted more and more attention from researchers [12].

Salinity can cause the expression of certain stress resistance genes in plants, and eventually lead to the hyperaccumulation of various metabolites in plants, and the changes of plant metabolites basically affect the salt tolerance of plants [13, 14]. The most common osmomodulatory substances that accumulate under salt stress include proline, hydroxyproline glycine betaine, polyamines, sugars and sugar alcohols [15, 16]. In addition, phenolic compounds, such as phenolic acids and flavonoids, are central to secondary metabolism and are increased in stressed plants [17]. The biosynthesis of phenolic cascades is attributed to their essential role as antioxidants responsible for scavenging stress-induced overproduction of free radicals, thereby counteracting stress caused by oxidative damage [18, 19]. In addition, phenolics have other secondary roles in mitigating the effects of stress, including regulating phytohormones and repairing stress-related photosynthetic apparatus damage [20].

Salt tolerance in plants usually respond to salt stress by reducing osmotic stress, ion toxicity, and oxidative stress [21, 22]. Wild barley responds to salt stress by activating ion transporters such as *NHX* (sodium exchanger), *AKT* (inward rectifying K +), *HKT* (high affinity potassium transporters) and *CAX* (vacuolar cation/proton exchanger) [23]. In recent years, there have been many studies using transcriptome and metabolome analysis techniques to analyze *kiwifruit* [24], *Sugar beet* [25], *rice* [26], and *Sorghum bicolor* [27]. Transcriptome technology can explore differentially expressed genes. Metabolomics technology can display changes in differential metabolites. The combination of the two technologies is more conducive to exploring the metabolic regulation mechanism of plants.

Because cotton is a widely grown cash crop in the world, which has strong adaptability to saline alkali land, it is used as a pioneer crop to improve saline-alkali land [28]. The salt tolerance of plants is the result of the co-regulation of metabolites in vivo and the interaction of upstream genes. At present, there have been in-depth studies on the salinity tolerance of cotton, mostly focusing on the physiological and transcriptional levels. However, there are relatively few studies on the combined analysis of metabolomics and transcriptome in cotton under salt stress. In particular, the analysis of the regulation mechanism of salt stress under different time periods is less. Therefore, This study aimed to conduct a joint analysis of cotton metabolites and genes under different periods salt stress, in order to discover the changes of complex metabolites and potential biomarkers in cotton under salt stress, and provide new clues for the metabolic regulation of cotton.

## Materials and methods

### Plant material and sampling preparation

The plant seeds used in the experiment was Zhong9807, a high salt-tolerant upland cotton genotype, it was provided by the subject of stress resistance identification of Cotton Research Institute, Chinese Academy of Agricultural Sciences. The formal identification of the plant material was undertaken by the corresponding author of this article (Professor Wuwei Ye). The plant material has been deposited at the gene bank of the Institute of Cotton Research, Chinese Academy of Agricultural Sciences, under the accession ID: xcy2399. After the seeds were depilated with sulfuric acid and disinfected with sodium hypochlorite, they were washed with distilled water for 5 times and planted in a 16 h/8 h day/night alternating growth box. Cotton seedlings were unified management. When the cotton seedlings reached three true leaves, 200 ml (400 m M) salt (NaCl) solution was used to irrigate them in the nutrition bowl. The treatment time was salt stress at 0 h (L0), 3 h (L3), 24 h (L24) and 48 h (L48). The sampling site was the second true leaf. At least 8 plants were sampled from a biological repeat at each time point. There were four independent biological replicates for each sample. The leaves were taken immediately placed in liquid nitrogen for 20 min and then stored at -80 ℃ for subsequent metabolomics, transcriptomics and other experiments.

### Metabolomics analysis

#### Sample extraction and UPLC conditions

The sample was extracted according to the method of Li et al. [29]. The freeze-dried leaf was crushed using a mixer mill with a zirconia bead for 1.5 min at 30 Hz. 100 mg powder was weighted and extracted overnight at 4℃ with 0.6 ml 70% aqueous methanol. Following centrifugation at 10, 000 g for 10 min, the extracts were absorbed and filtrated before UPLC-MS/MS analysis. The sequence of metabolome was conducted in Biomarker Technologies Co., LTD.

The sample extracts were analyzed using an UPLC-ESI–MS/MS system (UPLC, Shim-pack UFLC SHIMADZU CBM30A system, www.shimadzu.com.cn, MS, Applied Bio-systems 4500 Q TRAP, www.appliedbiosystems.com.cn/). The analytical conditions were as follows, UPLC, column, waters ACQUITY UPLC HSS T3 C18 (1.8 µm, 2.1 mm*100 mm). The mobile phase was consisted of solvent A, pure water with 0.04% acetic acid, and solvent B, acetonitrile with 0.04% acetic acid. Sample measurements were performed with a gradient program that employed the starting conditions of 95% A, 5% B Within 10 min, a linear gradient to 5% A, 95% B was programmed, and a composition of 5% A, 95% B was kept for 1 min. Subsequently, a composition of 95% A, 5.0% B was adjusted within 0.10 min and kept for 2.9 min. The column oven was set to 40 °C. The injection volume was 4 μl. The effluent was alternatively connected to an ESI-triple quadruple-linear ion trap (QTRAP)-MS.

#### Mass spectrometric analysis conditions

LIT and triple quadruple (QQQ) scans were acquired on a triple quadruple-linear ion trap mass spectrometer (Q TRAP), API 4500 Q TRAP UPLC/MS/MS System, equipped with an ESI Turbo Ion-Spray interface, operating in positive and negative ion mode and controlled by Analyst 1.6.3 software (AB Sciex). The ESI source operation parameters were as follows: ion source, turbo spray; source temperature 550℃; ion spray voltage (IS) 5500 V (positive ion mode)/-4500 V (negative ion mode); ion source gas I (GSI), gas II (GSII), curtain gas (CUR) were set at 50, 60, and 30.0 psi, respectively; the collision gas (CAD) was high. Instrument tuning and mass calibration were performed with 10 and 100 μmol/L polypropylene glycol solutions in QQQ and LIT modes, respectively. QQQ scans were acquired as MRM experiments with collision gas (nitrogen) set to 5 psi. DP and CE for individual MRM transitions were done with further DP and CE optimization. A specific set of MRM transitions were monitored for each period according to the metabolites eluted within this period.

#### Qualitative and quantitative analysis of metabolites

Metabolites were analyzed qualitatively and quantitatively by mass spectrometry using the public metabolic database. The detected metabolites were shown in the MRM metabolite detection multi peak map of the multi reaction detection mode. Each mass spectrum peak with different colors represents a detected metabolite. The characteristic ions of each metabolite were obtained through triple quadruple screening. The strength of the characteristic ions was obtained in the signal detector, and was integrated and corrected with multi-quantity software. The peak area of each chromatographic peak represented the relative content of the corresponding metabolites [29].

In order to test the repeatability of samples under salt stress, QC samples (mixed sample extracts) were inserted into every 10 test samples during the analysis. The accuracy and reproducibility of metabolite detection can be determined by overlapping display analysis of mass spectrometry tic of different QC samples.

#### PCA, PCC and OPLS-DA analysis

Unsupervised PCA (principal component analysis) was performed by statistics function p rcomp within R (www.r-project.org). The HCA (hierarchical cluster analysis) results of samples and metabolites were presented as heatmaps with dendrograms, while pearson correlation coefficients (PCC) between samples were caculated by the cor function in R and presented as only heatmaps. Both HCA and PCC were carried out by R package *p* heatmap. For HCA, normalized signal intensities of metabolites (unit variance scaling) were visualized as a color spectrum.

#### Differential metabolites selection

Significantly regulated metabolites between groups were determined by VIP > 1, *p* value < 0.05 and fold change > 1.2 or < 0.83. VIP values were extracted from OPLS-DA result, which also contained score plots and permutation plots, it was generated using R package Me-taboanalyst R. In order to avoid over fitting, a permutation test (200 permutations) was performed.

Identified metabolites were annotated using KEGG Compound database (http://www.kegg.jp/kegg/compound/), annotated metabolites were then mapped to KEGG Pathway database (http://www.kegg.jp/kegg/pathway.html) [30, 31]. Pathways with significantly regulated metabolites mapped to MSEA (metabolite sets enrichment analysis), their significance was determined by hypergeometric test’s *p*-values.

### Transcriptome analysis

#### RNA-Seq analysis and identification of differentially expressed genes

Transcriptome sequencing was performed using TruSeq PE Cluster Kit v4-cBot-HS (Illumina) to identify the changes in gene expression in the cotton during the different salt time. We re-analyzed the raw data. Raw sequencing reads were filtered by FASTP, and purified valid reads were mapped to the cotton genome of *Gossypium hirsutum* (http://ibi.zju.edu.cn/cotton/) using HISAT2 [32] (version 2.0.5) with default parameters. A total of 76.55 Gb of clean data were obtained. Q30, Q20 and GC content values were obtained after filtering and revealed that the sequencing data were high quality (Table S1). Align the Clean Reads of each sample with the designated reference genome, and the alignment efficiency ranges from 96.78% to 97.65% (Table S2). DESeq2 [33] was suitable for differential expression analysis between sample groups. We used fragments per kilobase of transcript per million mapped reads (FPKM) valued to represent gene expression levels. Based on the comparison results, alternative splicing prediction analysis, gene structure optimization analysis and discovery of new genes were performed, and 11,364 new genes were discovered, of which 8,990 were functionally annotated. The BLAST [34] software was used to compare sequence for discovered new gene with NR, Swiss Prot, GO, COG, KOG, Pfam, and KEGG databases [35, 36] by using an *e*-value cutoff of 10^–5^. The KOBAS 2.0 was used to obtain the KEGG Orthology results of the new gene. The amino acid sequence of the new gene was predicted, and HMMER software was used to compare with Pfam database to obtain annotation information of the new gene. Differentially expressed genes were identified using |fold change (FC) |≥ 2 with false discovery rate (FDR) < 0.01.

#### qRT–PCR analysis

RNA extraction was used EASYspin Plant RNA Kit (Aidlab Biotech, Beijing, China). RNA reverse transcription used TranScript*®* II All-in-One First-Strand cDNA Synthesis SuperMix for qPCR (One-Step gDNA Removal) from TransGen Biotech. 1000 ng RNA to reverse transcribe into cDNA, and diluted the cDNA four times for qRT-PCR. The qRT-PCR was performed by using the Gene Applied Biosystems*®* 7500 Fast and TransStart Top Green qPCR SuperMix (TransGen Biotech, Beijing, China). qRT–PCR was conducted in a 20 μl volume containing 1 μl of diluted cDNAs, 0.4 μl of the each primer, 0.4 µL of passive reference dye, 7.8ul H_2_O, and 10 µL of Top Green qPCR SuperMix under the following conditions: 95 ◦C for 300 s, followed by 40 cycles of 95 ◦C for 15 s, 58 ◦C for 20 s and 72 ◦C for 30 s. *GhActin* gene was used as an internal reference gene. The 2^−△△Ct^ method was used to calculate relative expression levels [37, 38].

#### Statistical analysis

The Graphpad Prism 8.0 software was used to plot charts. Significant differences among all the experimental treatments were determined using a one wey ananlysis of variance (ANOVA) followed by Tukey’s test by IBM SPSS version 21.0 software. Metware Cloud Platform, BMK Cloud Platform, and TBtools were used to draw heat maps. The merge images was used Adobe Photoshop CS6. Cytoscape 3.9.1 was used to draw correlation graphs.

## Results

### Phenotypic changes of cotton leaves under salt stress

**Fig. 1:**
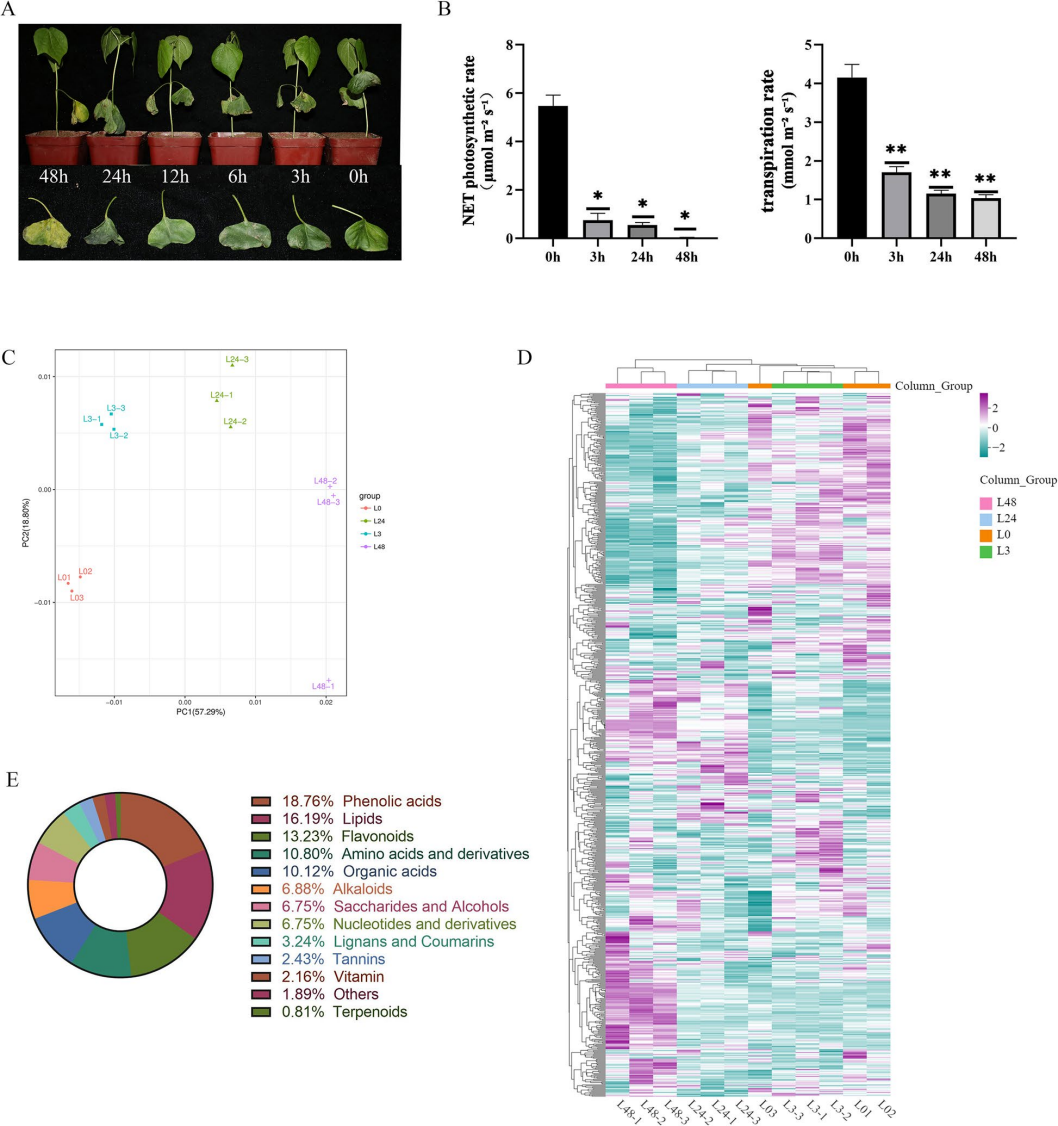
Comparative classification of phenotype and all metabolite of cotton under salt stress. **A** Phenotypic analysis of cotton under salt stress. **B** Analysis of net photosynthetic rate and transpiration rate. **C** Quality control of metabolomics data. **D** Heatmap showed the results of the clustering analysis of DMs. **E** Classification of metabolites under salt stress

The effect of salt stress on cotton leaves was obvious. As shown in Fig. 1A, cotton seedlings showed slight phenotypic changes after 3 h of salt irrigation, and manifested as mild dehydration of cotyledons by naked eye observation. When the plants were stressed for 3 to 12 h, the cotyledons became severely wilted, but the true leaf phenotype remained unchanged. Cotton seedlings were stressed to 24 h, the stem base of a single seedling was lodging, and the cotyledons wilted severely. At 48 h, the cotyledons turned yellow. The leaves had shrunk severely, the true leaves had dehydrated and wilted, and half of the cotton seedlings had broken and bent at the base of the stem. The net photosynthetic rate and transpiration rate of leaves decreased after salt stress, which indicated the salt stress affected the photosynthesis of leaves (Fig. 1B). These results showed that the damage of open salt treatment to cotton seedlings gradually increased with time, and photosynthesis was became more weaker.

### Metabolome analysis of cotton under salt stress

#### Quality control of metabolome data

In order to further understand the changes of metabolites at different time periods in cotton leaves under salt stress, the dynamic changes of metabolites in leaves were determined using a widely-targeted metabolomics method. Principal component analysis (PCA) was subsequently performed, and the results showed a significant separation between the L0 samples and the other three samples (Fig. 1C). Notably, both the L0 and L3 groups were located on the left side of the Y-axis, which was distinct from the L24 and L48 groups. Similarly, in the HCA analysis, the samples from the L0 and L3 groups were also clustered together (Fig. 1D). The orthogonal projections latent structures-discriminant analysis (OPLS-DA) was performed, and the score showed that different treatments also displayed significant segregation in the OPLS-DA results (Figure S1).

#### Metabolite analysis in cotton

**Fig. 2:**
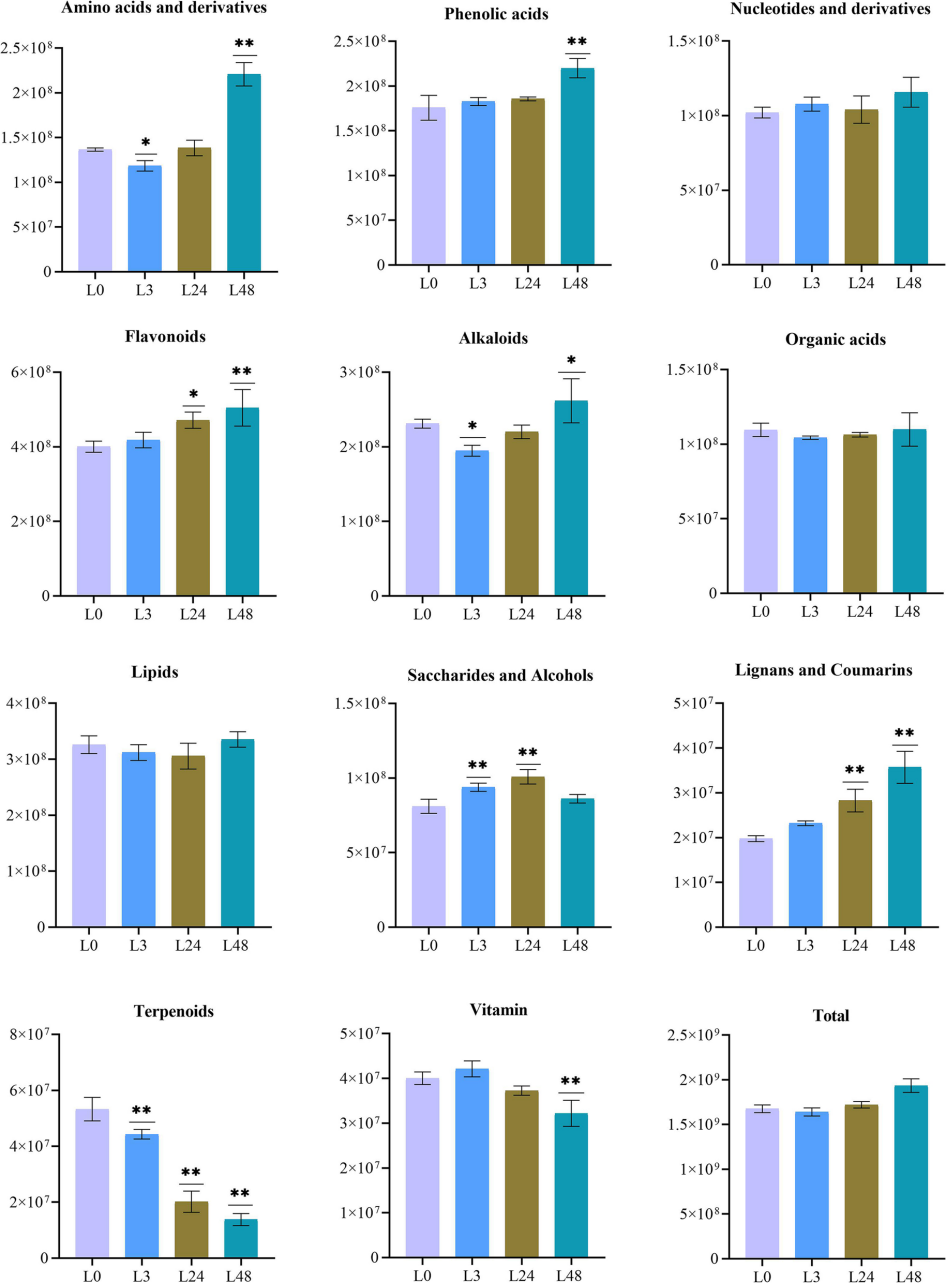
Analysis of total ion abundance of metabolite categories under NaCl stress

A total of 741 metabolites were identified across four time periods (L0, L3, L24, and L48) and grouped into 13 classes by widely-targeted metabolomics analysis (Fig. 1E). According to the metabolite classification results, there were 9 types of compounds that account for more than 80% of the total metabolites in leaves, including phenolic compounds (19%), lipid (16%), flavonoids (13%), amino acids and derivatives (11%), organic acid (10%), alkaloids (7%), nucleotides and derivatives (7%), saccharides and alcohols (7%). As shown in Fig. 2, the contents of flavonoids, lignans and coumarins were relatively high, but the contents of terpenes and vitamin were significantly reduced. The levels of lipids and organic acids decreased in the L3 and L24 group but increased in the L48 group. Conversely, saccharides and alcohols content also increased in the L3 and L24 group. In general, with the extension of salt stress time, the contents total metabolites showed a trend of first decreasing and then increasing. It can be seen that these substances may have a very important relationship with cotton salt stress.

**Fig. 3:**
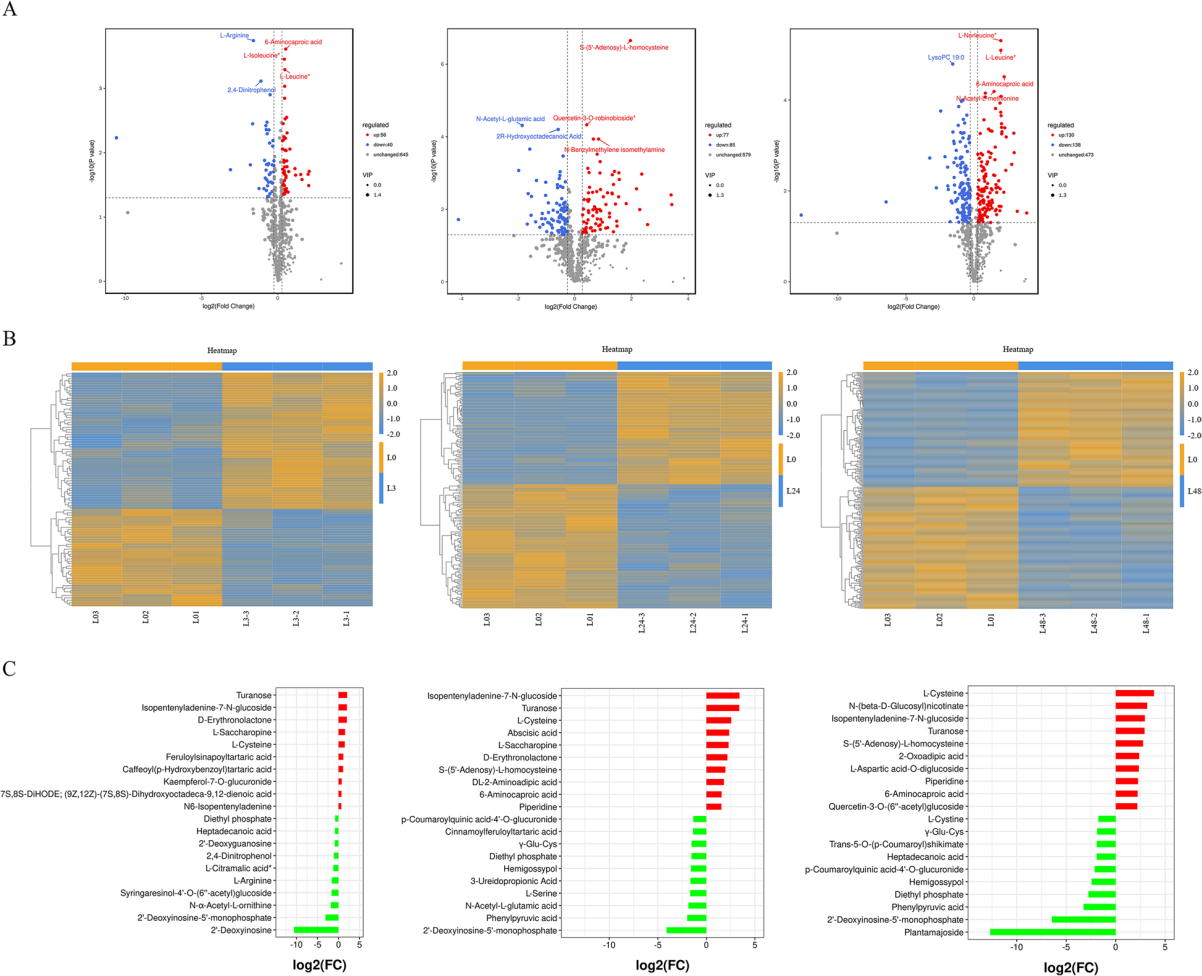
Up- and down-regulation of DMs in different treatment groups. **A** Volcano plots showing the up-regulate and down-regulated metabolites. **B** Heatmap showing the up- and down-regulated metabolites. **C** Up- and down-regulation of the top ten DMs

According to the volcanic map analysis(Fig. 3A), there were 96 differential metabolites (56 up-regulated and 40 down-regulated) in L0vsL3 group, 162 differential metabolites (77 up-regulated and 85 down-regulated) in L0vsL24 group, 268 differential metabolites (130 up-regulated and 138 down-regulated) in L0vsL48 group, 119 differential metabolites (57 up-regulated and 62 down-regulated) in L3vsL24 group, 169 differential metabolites (88 up-regulated and 81 down-regulated) in L24vsL48 group, 243 differential metabolites (114 up-regulated and 129 down-regulated) in L3vsL48 group. Amino acids (L-arginine, L-leucine, L-isoleucine, N-acetyl-l-glutamic acid, N-leucine, L-phenylalanine, N-acetyl-l-methionine, 6-aminoacetic acid, L-phenylalanine), lipid substances (2, 4-dinitrophenol), and flavonoids (quercetin-3-robinoside) had higher *p* values in the comparison of each group, indicating that they may play an important role under salt stress. A heatmap of DMs was constructed. The DMs in these three comparison groups clearly reflect the above mentioned changes (Fig. 3B). According to the change of the FC in the accumulation of the metabolites, we identified the top ten DMs that increased or decreased in each comparison group (Fig. 3C).

#### K-means cluster analysis of differential metabolites

**Fig. 4:**
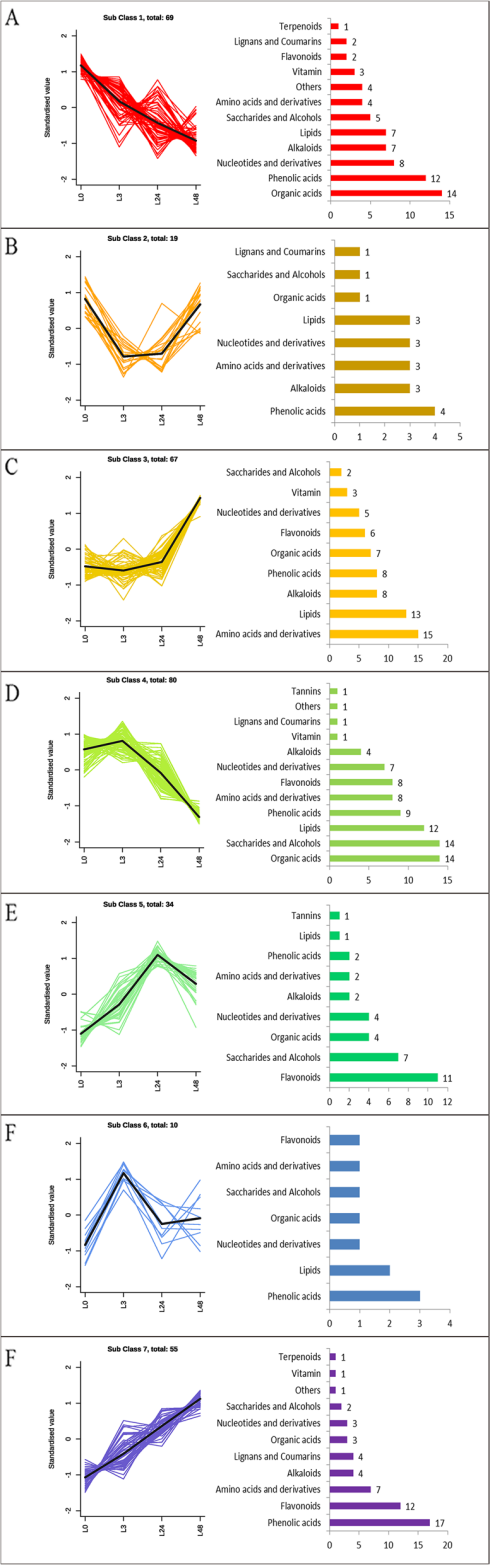
K-means cluster analysis of differential metabolites and their metabolite categories

In order to determine the change trend of different metabolites and different time under salt stress, the K-means time cluster analysis was carried out on 344 co-expressed metabolites (Fig. 4). The results showed that the trends could be divided into 7 categories. A total of 69, 19, 67, 80, 34, 10, 55 metabolites were clustered in 1 to 7 categories, respectively. Among them, with the extension of salt stress time, the expression trend of category 1 and 7 was that their content continued to rise or decline. The most different metabolites in class 1 were belonged to organic acids and phenolic acids. In contrast, most of the differential metabolites in the 7 subgroups belonged to phenolic acids and flavonoids.

**Fig. 5:**
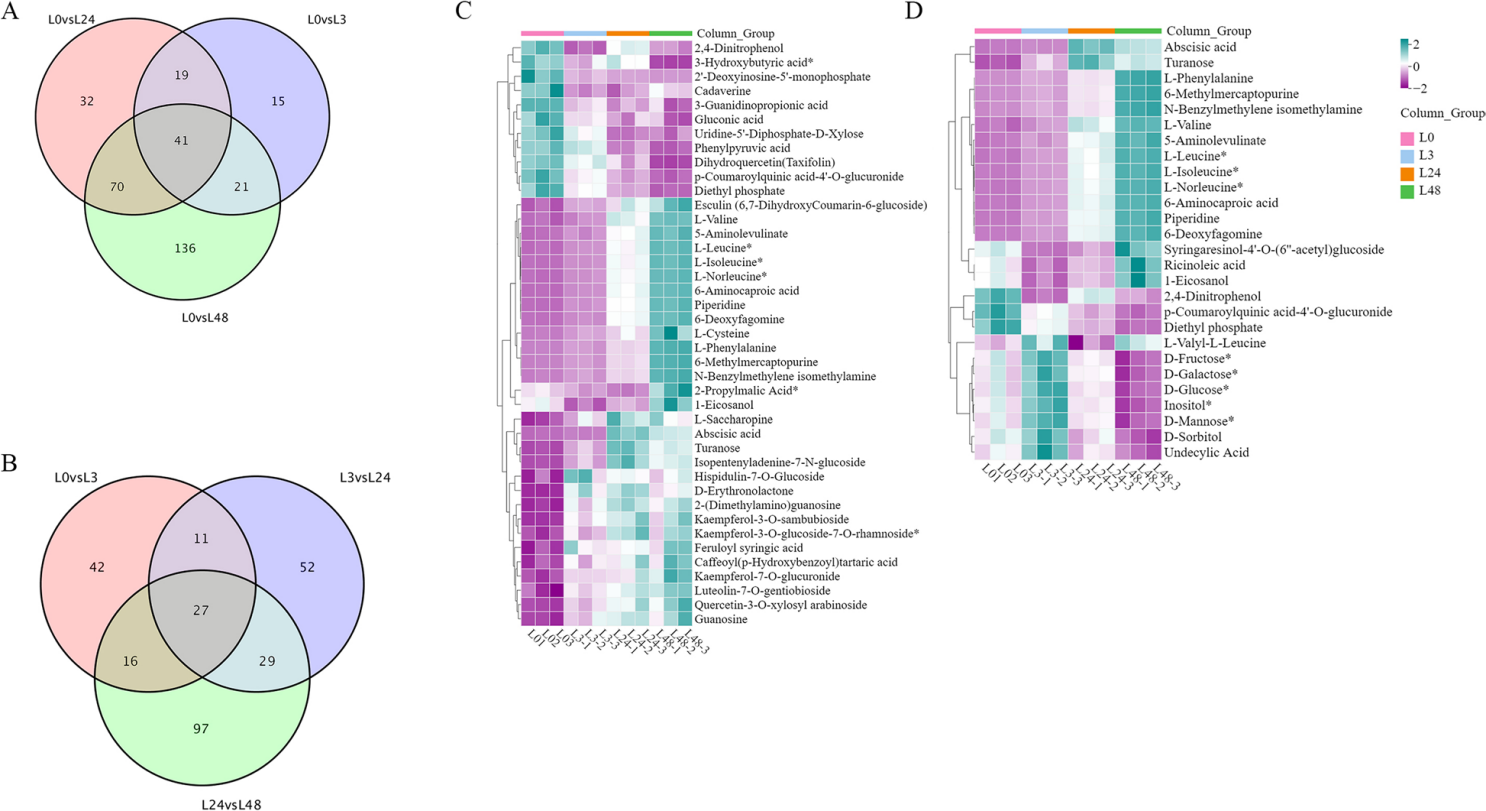
Differential metabolites analysis of cotton under salt stress. **A** Venn diagram of differential metabolites was shown in L0vsL3, L24, L48. **B** Venn diagram of differential metabolites was shown in L0vsL3, L3vsL24 and L24vsL48. **C** Heat map of common metabolites was displayed in L0vsL3, L24 and L48. **D** Heat map of common metabolites was displayed in L0vsL3, L3vsL24 and L24vsL48

The common and unique differential metabolites in these three periods were very important for the mining of salt tolerant metabolites in cotton. Therefore, we drew the venn diagram of metabolites in each time period, and obtained the co-enriched metabolites and unique differential metabolites between L0vsL3, L0vsL24 and L0vsL48 (Fig. 5). There were 41 common differential metabolites in the three comparison groups (Fig. 5A). Their accumulation patterns were shown in the Fig. 5C, which were mainly divided into five categories. The differential metabolites with increased content involve flavonoids and amino acids. At the same time, the co-enriched metabolites and unique differential metabolites were obtained between L0vsL3, L3vsL24 and L24vsL48 (Fig. 5B). There were 27 common differential metabolites compared in pairs in the four time periods. Their expression patterns were shown in the Fig. 5D, which were mainly divided into three categories. The differential metabolites with increased content involve lipids and amino acids.

#### KEGG enrichment analysis of differential metabolites

**Fig. 6:**
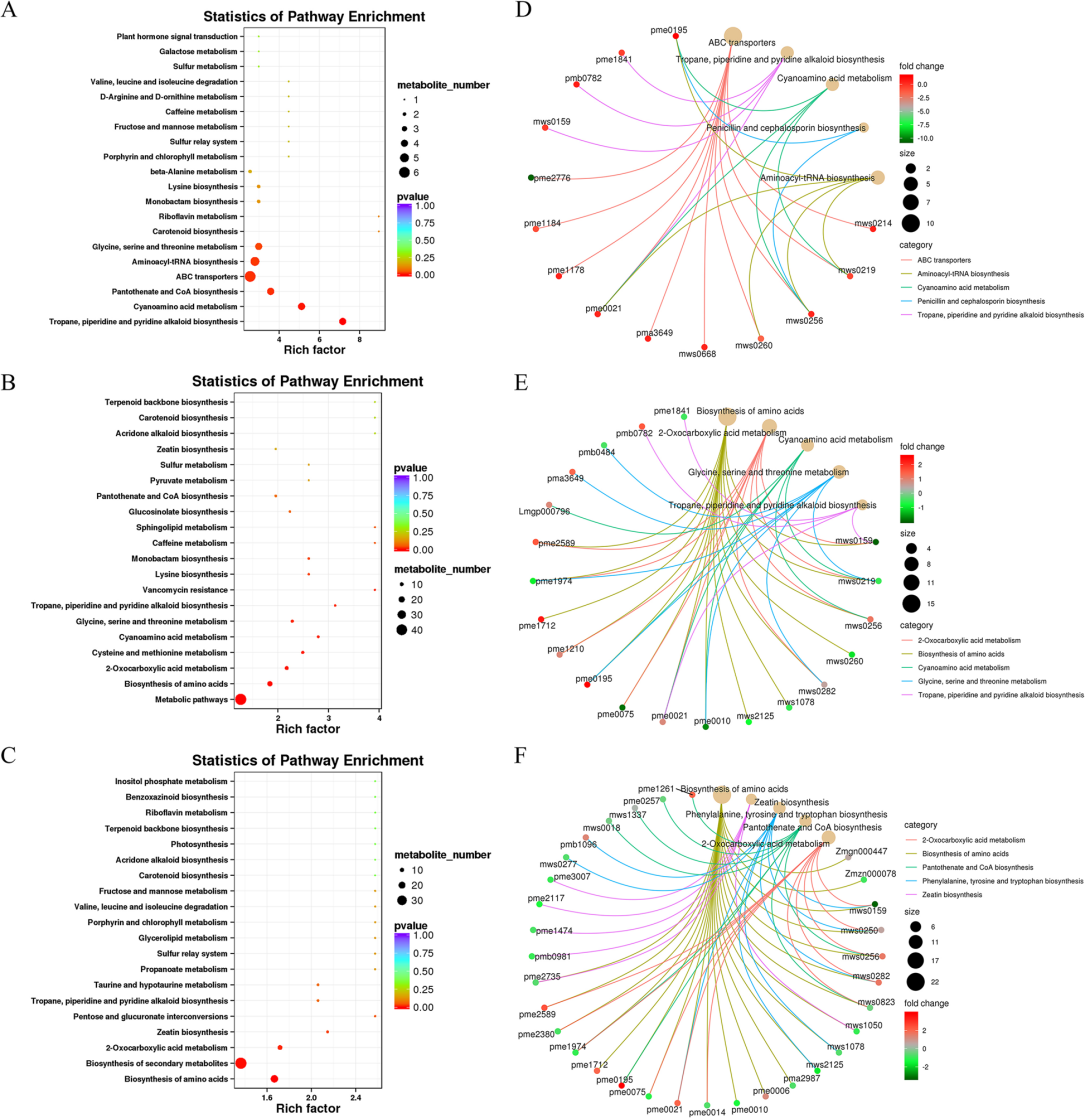
Differential metabolite enrichment pathways and their correlation analysis. **A**, **B**, **C**, differential metabolites were enriched KEGG pathway in L0vsL3, L0vsL24, L0vsL48 group. **D**, **E**, **F** the correlation analyzed the top five metabolic pathways and differential metabolites in L0vsL3, L0vsL24, L0vsL48 group

We classified the differential metabolites by KEGG annotation using the KEGG database, and found that the differential metabolites were mainly enriched in the synthesis of secondary substances and amino acid biosynthesis and metabolism (Fig. 6A, B, C). These differential metabolites were further subjected to KEGG enrichment analysis, and it was found that they were mainly enriched in primary metabolic pathways such as amino acids at the initial stage of salt stress. With the prolongation of salt stress time, the differential metabolites shifted from amino acid metabolism to secondary metabolism. We used correlation to represent the relationship between metabolic pathways and differential metabolites in the top 5 pathways significantly enriched by KEGG. In the comparison of these several times, we found that L-cysteine and L-arginine were actively involved these 5 metabolic pathways (Fig. 6D, E, F).

**Fig. 7:**
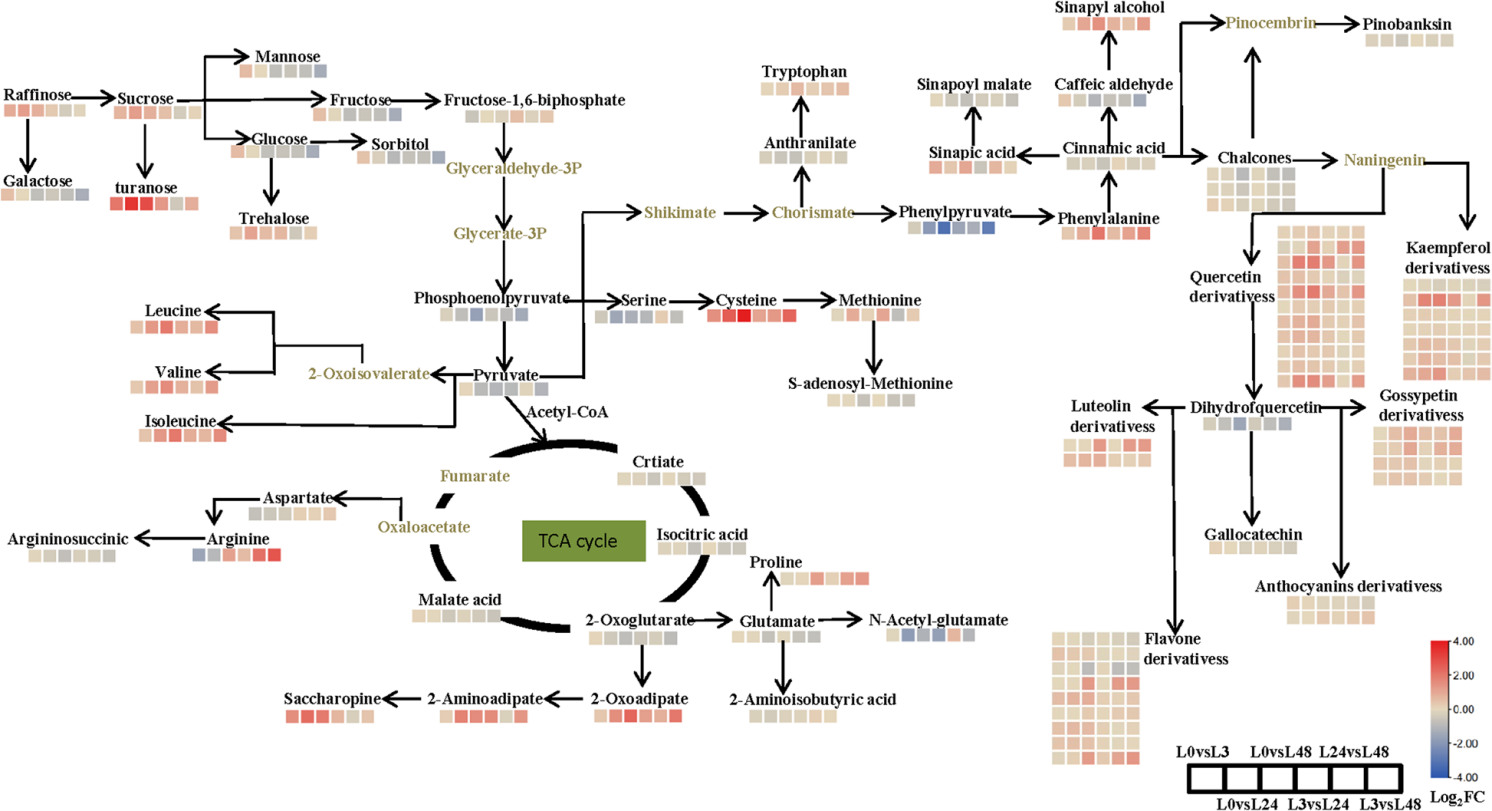
Several metabolic regulatory networks mainly involved in metabolites in the leaves of cotton under salt stress. The metabolites written in gray were not detected in this study. The differential metabolite changes were represented by the log_2_FC ratio. Blue represents a decrease in content and red represents an increase in content

In order to comprehensively understand the changes of metabolites under salt stress, we proposed the change process of primary and secondary metabolites in cotton leaves under salt stress on the basis of literature and web-based metabolic pathway database, the main pathways include glycolysis, TCA cycle, amino acid metabolism, and secondary metabolic pathways (Fig. 7). We identified 10 metabolites associated with the glycolytic pathway, including raffinose, sucrose, turanose, glucose, glucose-6-phosphate, fructose-6-phosphate, fructose 1, 6-bisphosphate and sorbitol. Turanose content was significantly increased at all time periods after salt stress compared to 0 h. There was also a significant increase in other sugars but not as much as turanose. Phosphoenolpyruvate was converted to pyruvate and shikimate, respectively. Pyruvate and acetyl-CoA entered the TCA cycle, and the metabolites involved in the TCA cycle were citric acid, isocitrate, fumaric acid, and 2-ketoglutarate, and these TCA cycle products all decrease significantly under salt stress. Proline and aminobutyric acid were synthesized by glutamic acid with a-ketoglutarate as precursor. Under salt stress, proline began to increase significantly at 24 h. The results showed that 24 h treatment could effectively stimulate the response of proline to salt stress in cotton leaves. In addition, it was also found that the metabolites of met pathway were involved in the response to salt stress, in which S-adenosyl methionine decreased at 24 h. Shikimic acid can be converted to phenylalanine and enter secondary metabolism. Under salt stress, phenylalanine content increased significantly from 24 to 48 h. Similarly, the contents of cinnamic acid, mustard malic acid and caffeic acid decreased significantly at 24 h. More phenolic acids were used to synthesize flavonoids, especially flavonol substances quercetin-3-O- (6''-acetyl) glucoside, quercetin-3-O- (6''-malonyl) galactoside, kaempferol-3-O-(6''-malonyl) galactoside, quercetin-7-O-(6''-malonyl) glucoside, kaempferol-7-O-glucuronide, and they may play an important role in scavenging ROS.

### Transcriptomic analysis

#### Differentially expressed gene recognition and analysis

**Fig. 8:**
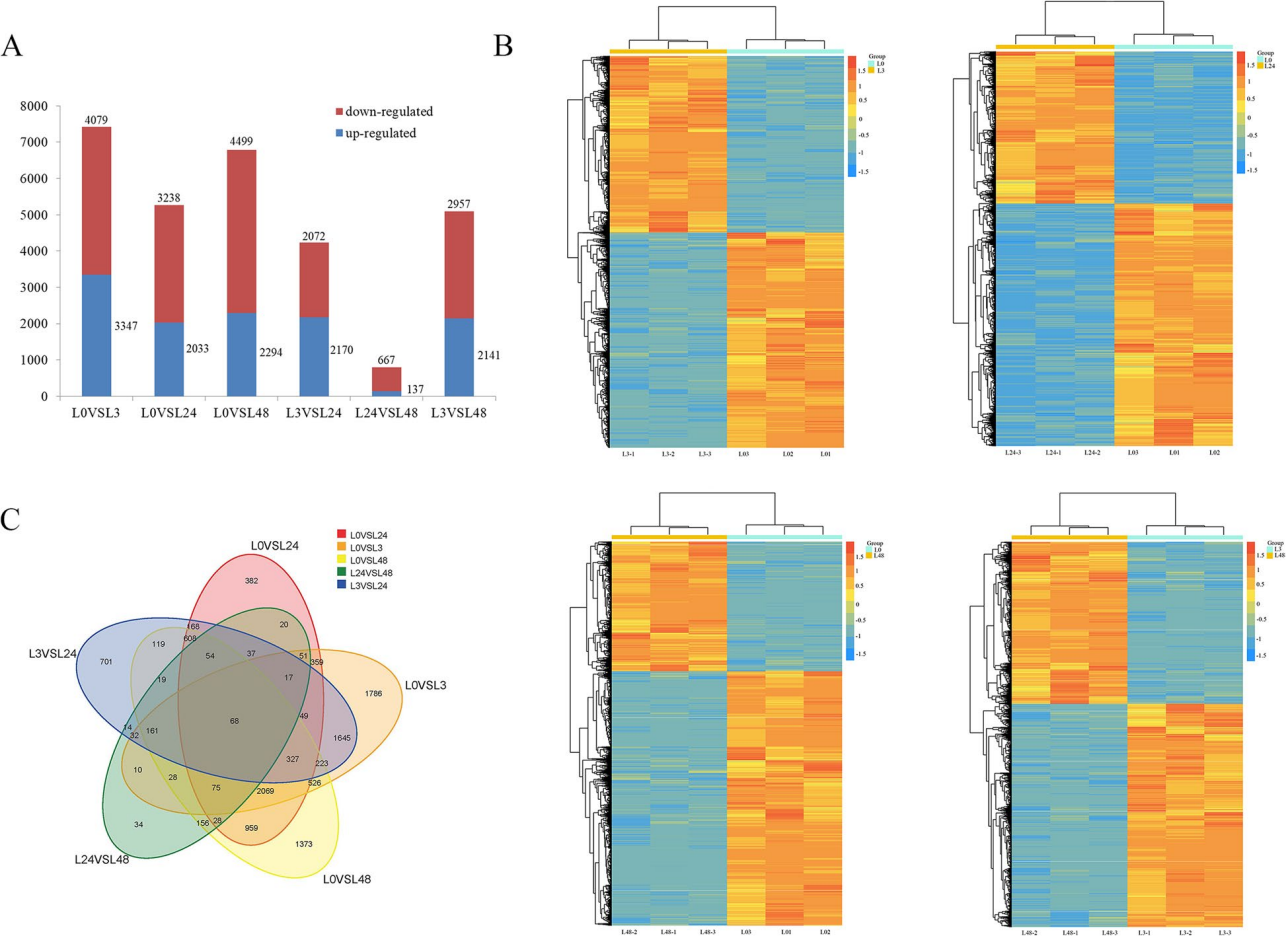
Changes in DEG expression. **A** Up-regulation and down-regulation of DEGs. The red box represents down-regulation, and the blue box represents up-regulation. **B** Heatmaps of DEGs compared between different groups. **C** Co-expression of DEGs in all comparison groups

The distribution of DEGs in the different treatment groups was shown in Fig. 8A. We compared the up-regulated and down-regulated DEGs in the L0 treatment group with other salt time treatments. The results revealed 7426 (3347 up-regulated and 4079 down-regulated), 5271 (2033 up-regulated and 3238 down-regulated) and 6793 (2294 up-regulated and 4499 down-regulated) DEGs in the three comparison groups. The largest number of DEGs was detected in the L0vsL3 comparison. The same results were shown in a heatmap (Fig. 8B). Subsequently, 68 DEGs were identified in cotton treated with all the different salt time (Fig. 8C). Analysis of ion transport related genes under salt stress has been conducted, and significant differences in the expression of *NHX*, *CIPK*, *AKT*, *CML*, and *NRT* related genes have been found (Figure S3).

#### K-means cluster analysis of differentially expressed genes

**Fig. 9:**
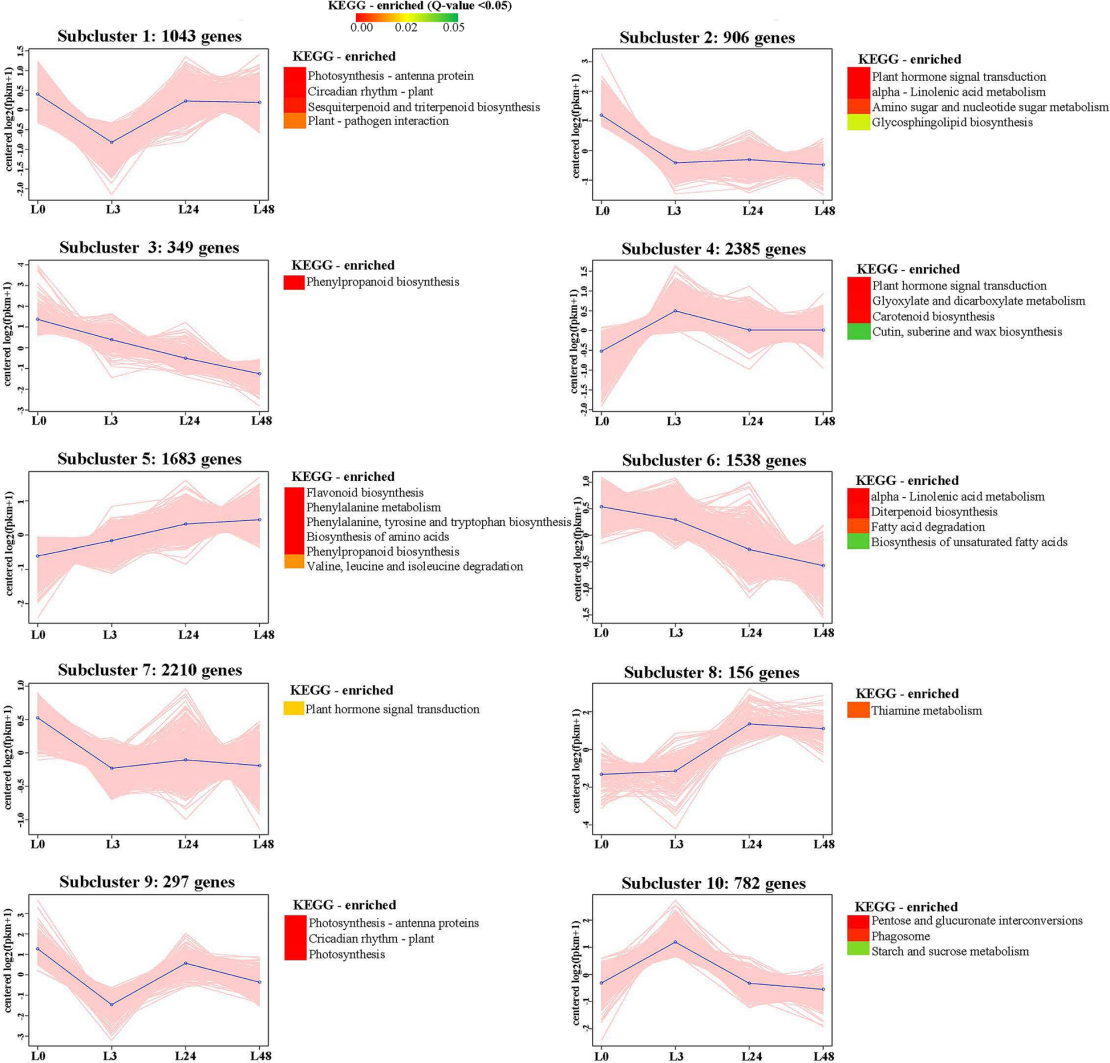
K-means clustering of gene expression profiles in cotton during salt stress

To study the genes expression patterns in different time of salinity conditions, a K-means cluster analysis was performed. A total of 11,349 genes were identified for K-means cluster analysis between L0vsL3, L0vsL24 and L0vsL48 comparison groups. These differentially expressed genes were classified into 10 clusters by K-means cluster analysis (Fig. 9). The DEGs of each cluster were analyzed for KEGG enrichment, and the KEGG enrichment metabolic pathway with Q value < 0.05 was screened. Genes showed different or the same change trend at different times under salt stress. For example, The subcluster 5 was contained 1683 DEGs, which showed significantly up-regulated in the four time periods under salt stress, and the differential expressed genes were significantly enriched in flavonoid pathway and amino acid metabolic pathway, indicating that these genes and pathways have been playing a role in coping with salt stress. Subcluster 3 and 6, just contrary to the trend of the subcluster 5, the differential expressed genes were continuously down regulated, and these genes were enriched in α-Linolenic acid metabolism, phenylpropionic acid biosynthesis, diterpene biosynthesis, fatty acid degradation and unsaturated fatty acid biosynthesis, and showed that salt stress significantly inhibited the expression of these genes. We found that subcluster 1 and subcluster 9 had the same time trend of gene expression, which were related to photosynthetic phosphorylation antenna protein, circadian rhythm and other metabolic pathways. The expression of these differential genes decreased after reaching the peak 24 h after salt stress, indicating that salt stress significantly affected the photosynthesis of cotton leaves. The differential genes of subcluster 4 and 10 had the highest expression after 3 h of salt stress, and then decreased. These genes were mainly concentrated in the metabolic pathways such as carotenoid, starch and sucrose metabolism. Interestingly, DEGs in the hormone signal transduction pathway were first down-regulated and then up-regulated, as shown in subcluster 2 and subcluster 7, indicating the diversity of plant hormone expression under salt stress. DEGs related to thiamine metabolism were also significantly up-regulated and enriched under salt stress, such as subcluster 8.

#### The qRT-PCR verification of gene expression

In order to confirm the accuracy of the genes obtained by RNA-Seq, we randomly selected 15 DEGs (Figure S2). They were detected by qRT-PCR. The similar expression trend of selected DEGs was consistent with that of illumina sequencing, indicating the reliability of RNA-Seq data. The detailed primer information was shown in Table S3.

#### Joint analysis between differential genes and differential metabolites

**Fig. 10:**
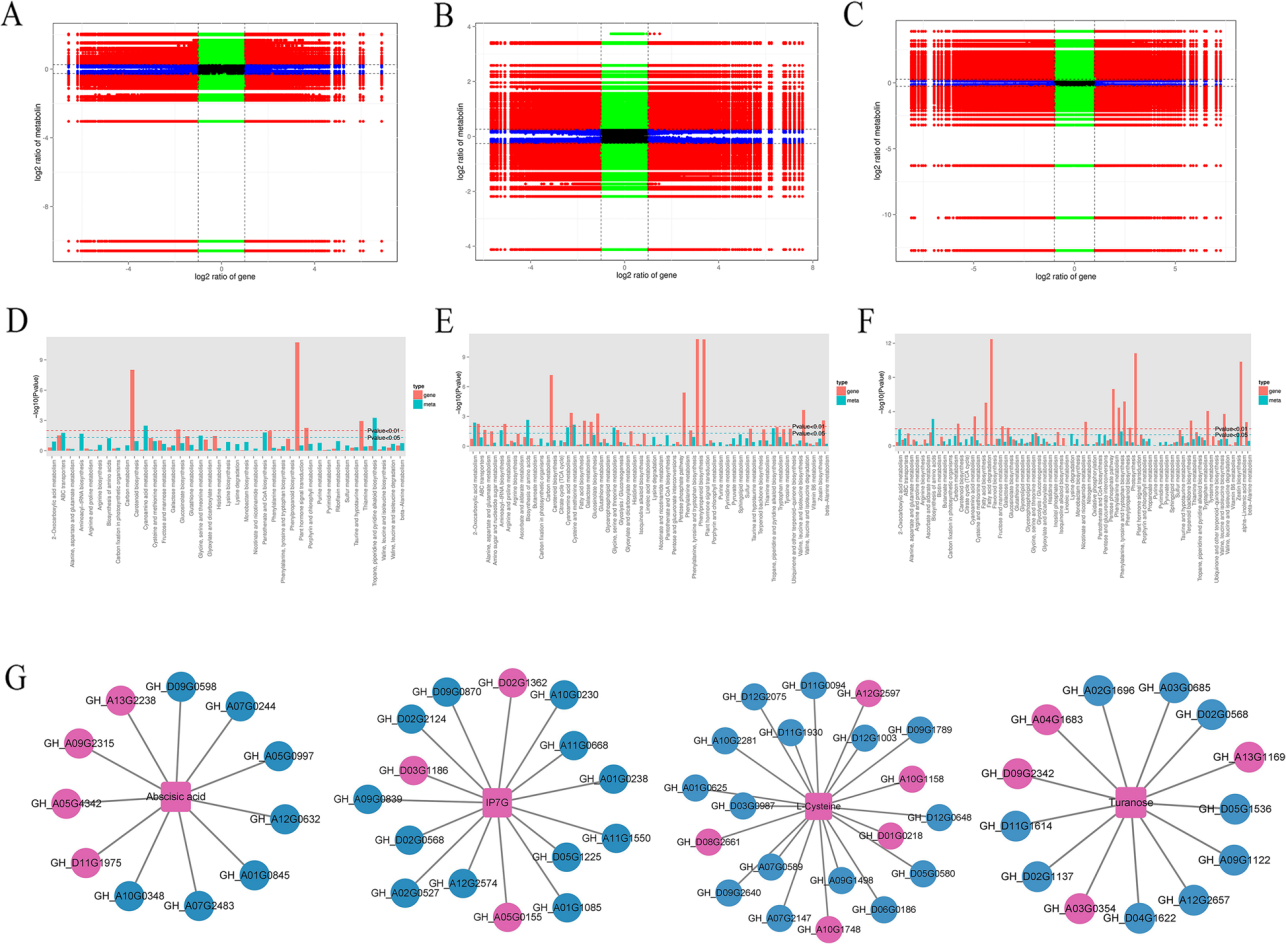
Correlation analysis of transcriptomic and metabolomic data from cotton exposed to different time of NaCl stress. **A**, **B**, **C** The nine-quadrant diagram shows the correlation of genes and compounds between the L0vsL3, L0vsL24 and L0vsL48 groups. **D**, **E**, **F** The DEGs and DMs enriched in the same KEGG pathway. **G** The genes with the highest correlation coefficient (PCC) related to hub metabolites, the blue color represented down-regulated, the pink color represented up-regulated, the round represented DEGs, and the square represented DMs

We performed a combined transcriptomic and metabolomic analysis to further understand the mechanism of cotton response to salt stress at different times. The correlation analysis revealed an increasing number of genes showing strong correlations with metabolites in L0vsL3, L0vsL24 and L0vsL48 comparisons (R > 0.8) (Fig. 10A, B, C). These results suggest that changes in the accumulation of these metabolites may be directly or indirectly regulated by the corresponding genes.

The DEGs and DMs of KEGG enrichment analysis showed that comparisons of L0 vs L3, L0 vs L24 and L0 vs L48 enriched many metabolic pathways, respectively (Fig. 10D, E, F). Among them, ABC transporters are significantly enriched in L0vsL3, and ABC transporters are responsible for the transport of amino acids, which indicates that amino acids are reprogrammed in the early stage of salt stress. L-cysteine and methionine biosynthesis were significantly enriched in the L0vsL24 comparison group. Amino acid biosynthesis, phenylalanine, tyrosine and tryptophan biosynthesis, these two pathways were significantly enriched in the L0vsL48 comparison group. On the basis of comprehensive analysis, cysteine and methionine biosynthesis, carbohydrate metabolism, plant hormone synthesis, carotenoid, phenylalanine and flavonoid metabolism changed significantly under salt stress.

The DEGs and DMs of 48 h were analyzed by PCC (*p* > 0.95) method, and it was found that abscisic acid, IP7G, turanose, and L-cysteine were associated with many differential genes(Fig. 10G). The abscisic acid was highly correlated with the transcription factor *MYB62*(GH_D09G0598), Zin finger (GH_A13G2238), Salicylate carboxymethyltransferase (GH_A12G0632). The IP7G was highly correlated with the glucan endo-1,3-beta-glucosidase (GH_A09G0839), cellulose synthase (GH_A12G2574). The L-cysteine was highly correlated the *ERF* (GH_D12G1003, GH_D06G0186), *NECD* (GH_D08G2661). The turanose was highly correlated the cytochrome P450 (GH_A09G1122, GH_A04G1683), and laccase-3 (GH_A03G0354). These results showed that the cytochrome may play an important role under salt stress.

## Discussions

Cotton is classified as a moderately salt tolerant crop with a salinity threshold level of 7.7dS m^-1^ [39]. Yet, salinity is also one of the most damaging environmental stresses on cotton [40]. Cotton was more susceptible to salt stress at germination, emergence and seedling stage than at other stages [41]. Cotton is recognized as a major crop in the world, one of the most important fiber crops and an important oil crop [3]. Salt damage to cotton stimulates a complex network of cells and molecules in the body to avoid injury and provide defense while maintaining growth and yield [42]. As the salinization of soil becomes more and more serious, it is particularly important to study the transcriptional and metabolic regulatory network of cotton under salt stress.

The effects of salt stress on plant physiological processes have been widely reported [43]. The conclusions we have reached corroborate these phenotypes. We analyzed the phenotypic characteristics of cotton under salt stress in four time periods. The results showed that the phenotypic changes of cotton leaves became more and more obvious from 0 to 48 h, which indicated that salt stress seriously affected the metabolism of cotton leaves. In our study, we used NaCl stress concentration of 400 m M, which was already a high salinity stress. However, cotton seedlings only showed serious wilting, which indicated that Zhong9807 was tolerant to salinity. This also proved that Zhong9807 was a salt tolerant genotype.

Amino acid metabolic pathways play an important role in protein biosynthesis, which components of several biosynthetic pathways, and it involved in signal transduction processes during plant stress responses. Amino acid metabolism also plays an important role in the primary and secondary metabolism of plants. Some amino acids are involved in nitrogen source assimilation and source-sink transport, while others are precursors to metabolites. Amino acid metabolism was significantly enriched in *Nitraria sibirica* Pall. under salt stress [44]. In this study, the amino acid metabolism pathways of cotton leaves were greatly enriched, which indicated that the harm of NaCl to cotton may be reduced by regulating amino acid metabolism, and it also proves that amino acid metabolism was closely related to salt stress.

In addition to its structural role in proteins, cysteine acts as a precursor to the metabolism of essential biomolecules vitamins, cofactors, antioxidants such as glutathione, and many defense compounds such as glucosinolates body [45]. Cysteine is not only the end point of sulfur metabolism, but also the starting point for the synthesis of various sulfur metabolites such as methionine, glutathione, and phytochelin, and is a key sulfur-containing compound. When plants were subjected to environmental stresses including heavy metals, chemical toxicity and salt stress, endogenous cysteine would increase [46–49]. At 200 m M NaCl, poplar leaves were more inclined to start the H_2_S cysteine cycle, maintain its speed, and activate the AOX pathway downstream, rather than shunting cysteine to methionine biosynthesis to burst ethylene. At 400 m M NaCl, more cysteine branches synthesize met, which was used to produce ethylene and induce AOX pathway to resist high salinity [50]. In plants, the first stage of cysteine synthesis is the conversion of serine to O-acetylserine (*OAS*) by serine acetyltransferase (*SAT*). Then, sulfide and *OAS* are converted to cysteine by OAS-TL, and cysteine can be degraded to pyruvate, ammonia and hydrogen sulfide by *CDE*, thus forming a cellular H_2_S-Cys cycle system in plants. The H_2_S-Cys cycle enhanced by salinity had a positive effect on the tolerance to salt stress, which was related to the manipulation of antioxidant and redox defense systems. Our results show that the content of cysteine in all amino acids was continuously up-regulated in different time periods of salt stress, and fluorescence quantitative verification showed that the key enzyme of cysteine synthesis and serine acetyltransferase were also up-regulated under salt stress (Figure S2). This also showed that cysteine plays an important role in cotton coping with salt stress. Cotton may form a new sulfur cycle system to cope with salt stress through cysteine.

Flavonoids are an important class of secondary metabolites in plants and are polyphenols with strong antioxidant capacity [51]. The content of total flavonoids in *kiwifruit* leaves was higher than that in fruit [52]. Flavonoids include flavonoids, dihydroflavonoids, dihydroflavonols, flavanols, chalcones, isoflavones, flavonols, anthocyanins, flavonoid carbon glycosides, dihydroisoflavonoids. The fruits of two *kiwifruit* varieties contain 118 flavonoids, more than half of which are glycosides, and these substances were mainly affected by flavonoid related structural enzyme genes [53]. When plants are under stress, flavonoids are produced in the body to combat abiotic stress. For example, kaempferol can absorb ultraviolet rays and reduce radiation damage to plants [54]. Accumulation of flavonoids such as kaempferol, quercetin, anthocyanins and procyanidins can enhance plant tolerance to salt stress [55, 56]. Chrysin, Rutin, and Daidzein in kiwifruit can could reduce serum levels of UA, BUN, Cr, and GAPDH in mice to varying degrees [57]. Flavonoids can improve plant adaptation to stress by eliminating ROS accumulation, or cooperate with other stress response factors such as ABA and GA to mitigate damage caused by unfavorable biotic or abiotic factors [58–60]. In this paper, the combined analysis of transcriptome and metabolome data found that flavonoids had significant dynamic changes at both 24 and 48 h of salt stress, and flavonoid pathway-related genes were significantly altered up at both 24 and 48 h of salt stress. Dihydroquercetin and catechin were decreased among flavonoids, but flavonols such as kaempferol-3-O-sambubioside, plantain-7-O-glucoside, luteolin -7-O-gentiobioside, quercetin-3-O-xylosylarabinoside, kaempferol-7-O-glucuronide, kaempferol-3-O-glucoside-7-O- Rhamnoside, and quercetin-3-O-rutin (rutin) were significantly increased, indicating that cotton undergoes a significant shunting of flavonoid metabolism under salt stress to cope with salt stress.

Through the time series accumulation trend of metabolites under salt stress, we found that the accumulation of carbohydrates and amino acids changed greatly in the early stage of salt stress, and the accumulation of flavonoids in the later stage of salt stress changed greatly. It shows that cotton responds to salt stress through energy metabolism and osmotic metabolism in the early stage of salt stress, and requires antioxidants such as flavonoids to cope with salt stress in the later stage of oxidative stress.

**Fig. 11:**
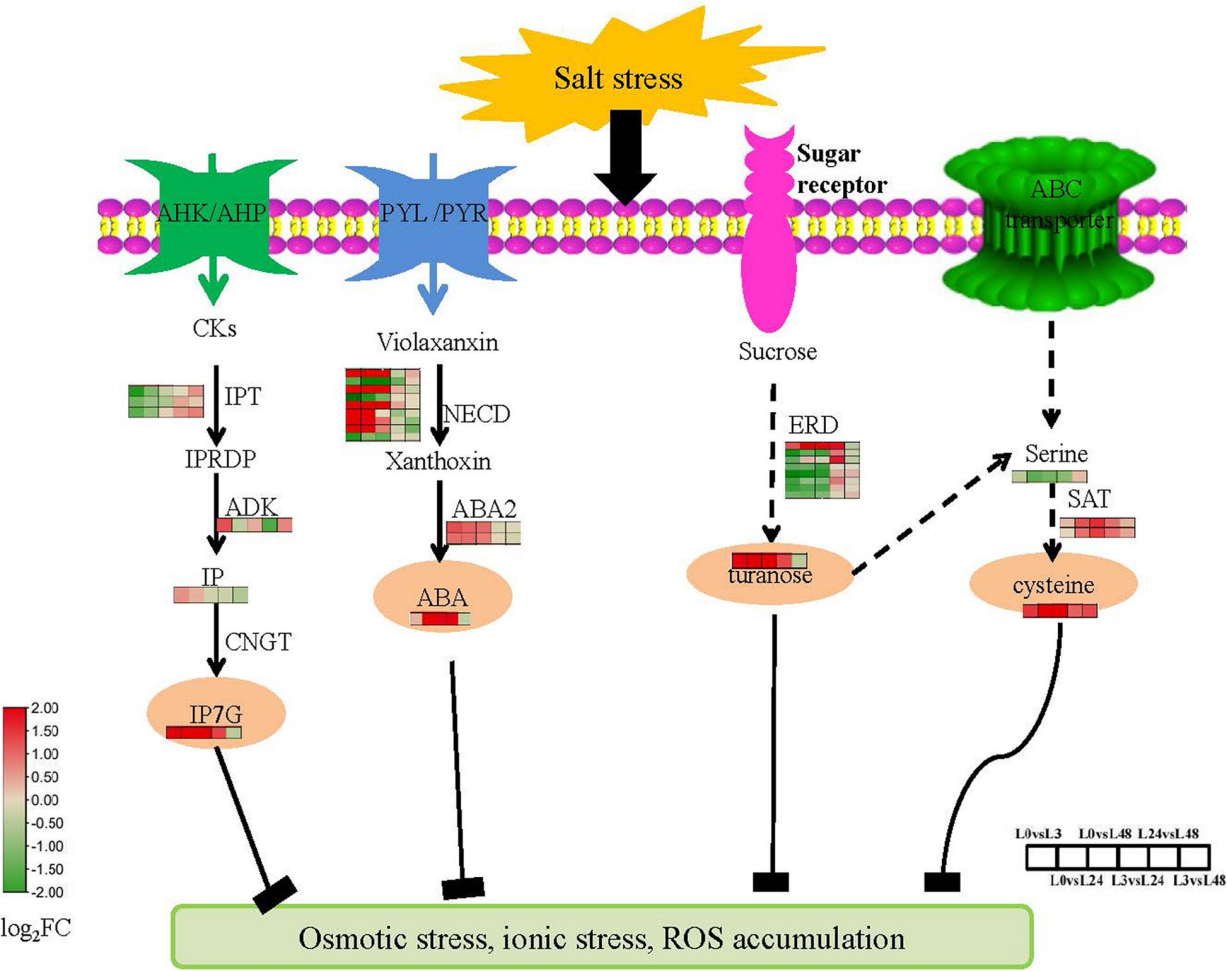
Metabolic regulatory network of major potential metabolites in cotton seedling resistance to salt stress

Mining candidate marker metabolites under salt stress is an important method to improve crop quality. Turanose is an isomer of sucrose, which naturally exists in honey. Turanose had anti-inflammatory effect in vitro [61]. It was a disaccharide produced by sucrose under the catalysis of sucrose amylase. Sugars provide energy for plants. Turanose content continued to rise in different time periods of salt stress. As one of the sugars, turanose is one of the potential biomarkers of cotton under salt stress. Transcriptome data showed that sucrose transporter and sucrose amylase genes were significantly up-regulated under salt stress, which may play an important role in the synthesis of turanose. ABA is a key plant hormone, which plays a key role in abiotic stress [62]. As one of the indicators of stress tolerance, ABA plays a key role in salt stress. Isopentenyladenine-7-N-glucoside is a cytokinin glycosylated salt organism, which also plays an important role in abiotic stress [63]. In this study, we identified 4 metabolites in cotton leaves as key salt tolerance biomarkers based on the metabolic pathways of DMs and expression of DEGs in the pathways (Fig. 11), which can be further validated in a large sample of cotton varieties. These metabolites were cysteine (Cys), Abscisic Acid(ABA), Isopentenyladenine-7-N-glucoside (IP7G), turanose. However, their accumulation trends were not consistent during the salt stress period, indicating that cotton had a complex salt tolerance mechanism.

## Conclusions

Based on the widely targeted metabolomics and transcriptome, osmotic stress and energy metabolism first occurred in cotton, and then oxidative stress was dominant in cotton under salt stress. Salt stress significantly affected the expression change of gene transcription level and metabolite accumulation in cotton leaves. The contents of amino acids, sugars and alcohols, lignans and coumarins, flavonoids and polyphenols increased in proportion during the whole salt stress period. In contrast, nucleic acids, lipids, alkaloids, organic acids and their derivatives and vitamins decreased significantly due to high salt stress. A total of 4 salt responsive metabolites represent potential biomarkers of salt stress tolerance in cotton. Specifically, we found that the synthetic genes realted to these four substances also changed significantly, which may be an important mechanism of salt tolerance in Zhong9807. In the future, functional genomics should be used to characterize these metabolites and their related genes, and validate the gene with the highest correlation coefficient (PCC) associated with hub metabolites. In conclusion, we provide some broad metabolic insights on salt stress tolerance and biomarkers of key metabolites, which may help to improve the tolerance of cotton to salt stress.

## Supplementary Information

**Additional file 1.** 

## Data Availability

The raw RNA-seq datasets supporting the conclusions of this article are available in the National Center for Biotechnology Information Bioproject repository, accession number: PRJNA623201. The reference genome of Gossypium hirsutum is available at http://ibi.zju.edu.cn/cotton/.

## Abbreviations

Cys: Cysteine
IP7G: Isopentenyladenine-7-N-glucoside
ABA: Abscisic acid
UPLC-MS/MS: Ultra-performance liquid chromatography/tandem mass spectrometry
MRM: Multiple reaction monitoring
QC: Quality control
PCA: Principal components analysis
OPLS-DA: Orthogonal projections to latent structures discriminant analysis
ANOVA: Analysis of variance
VIP: Variable importance in projection
DMs: Differentially accumulated metabolites
DEGs: Differentially expressed genes
