# Supplementary material for: Combined transcriptomic and metabolomic analyses elucidate key salt-responsive biomarkers to regulate salt tolerance in cotton
Source: BMC Plant Biol. 2023 May 10;23:245. doi: 10.1186/s12870-023-04258-z (PMC10170727; doi:10.1186/s12870-023-04258-z)
Supplement: Supplementary file 1 — Additional file 1. [file 12870_2023_4258_MOESM1_ESM.docx]

**Figure S1** OPLS analysis of metabolites

**
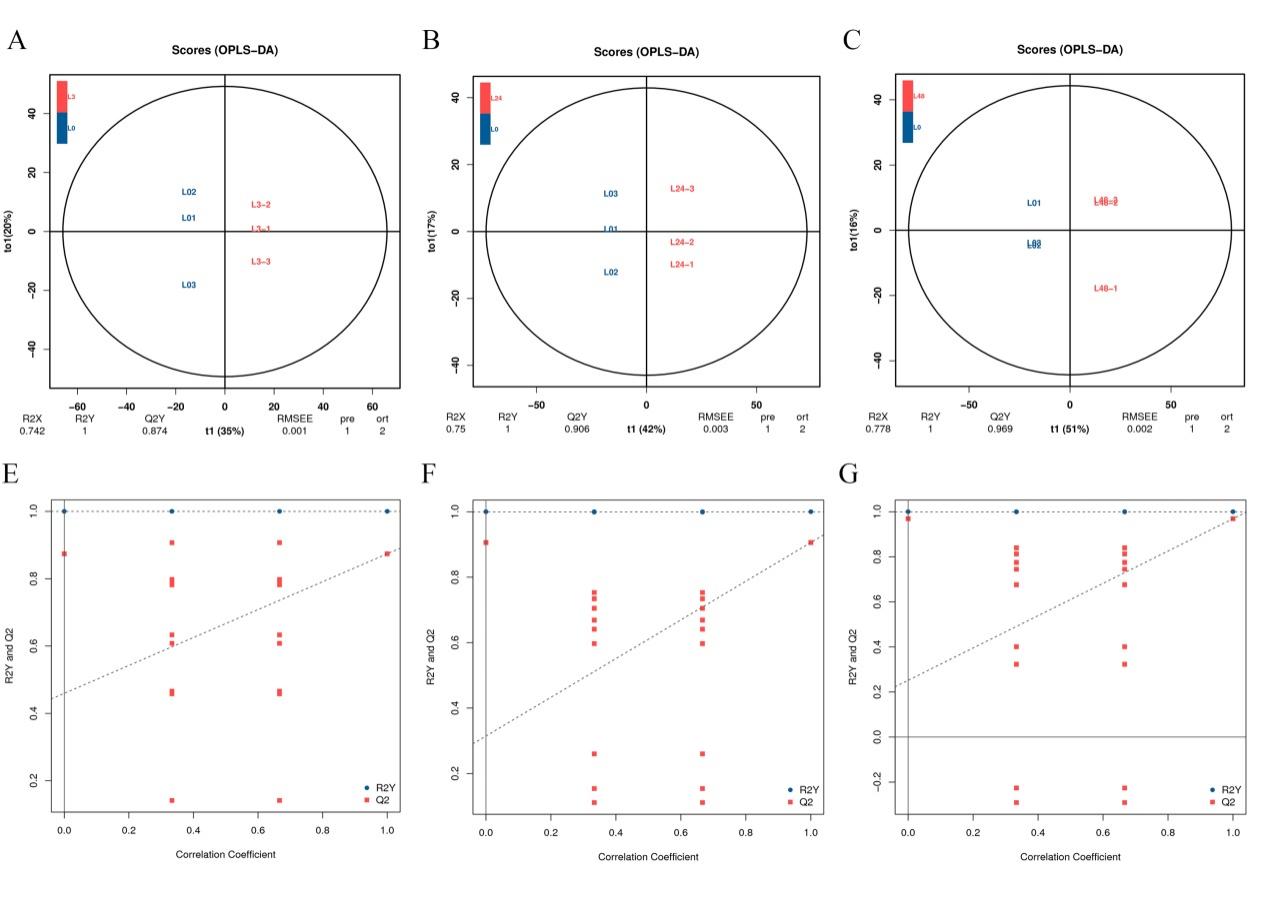
**

**Figure S2** Correlation analysis between qRT-PCR and RNA-Seq data

**
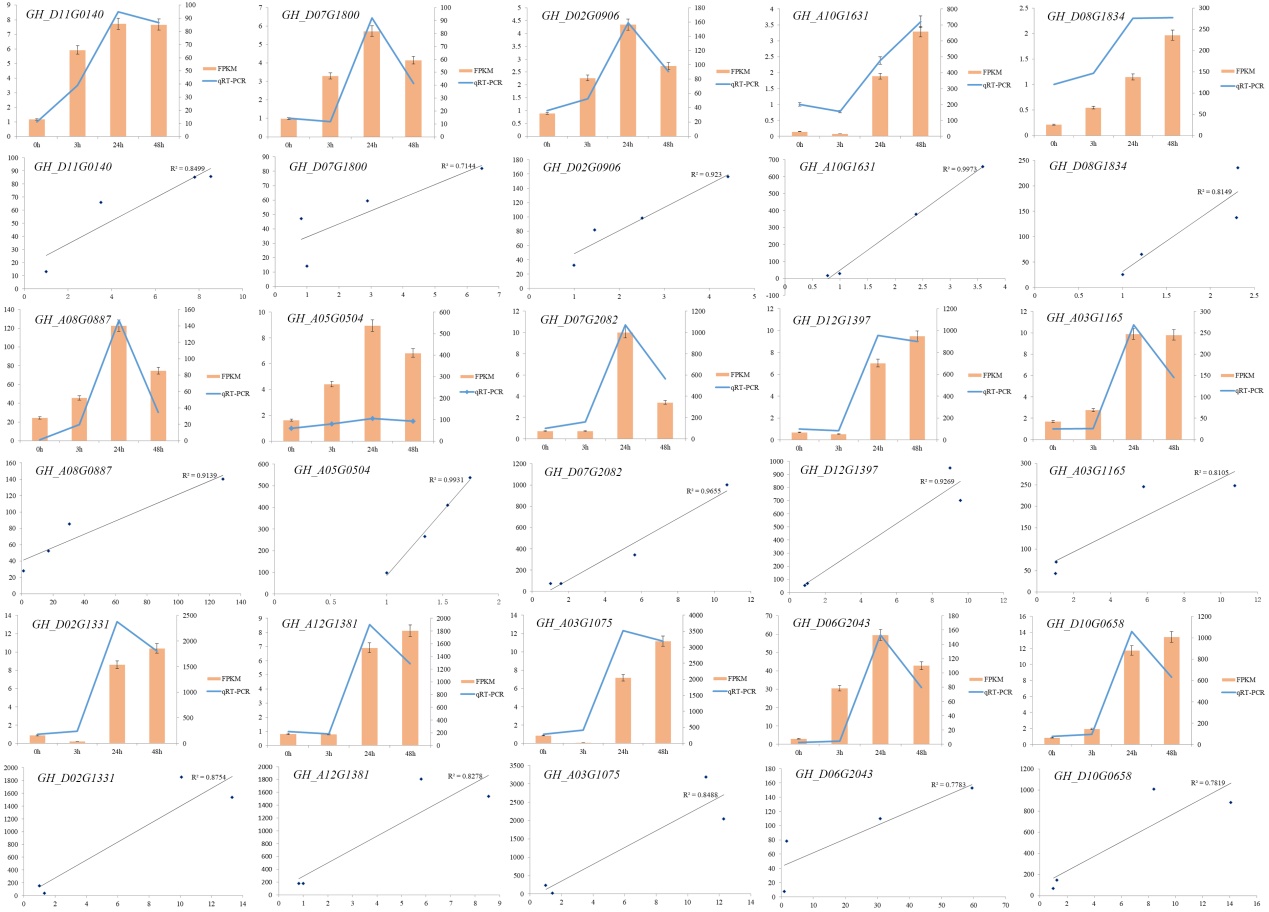
**

**Figure S3** The expression level of ion transport related genes

**
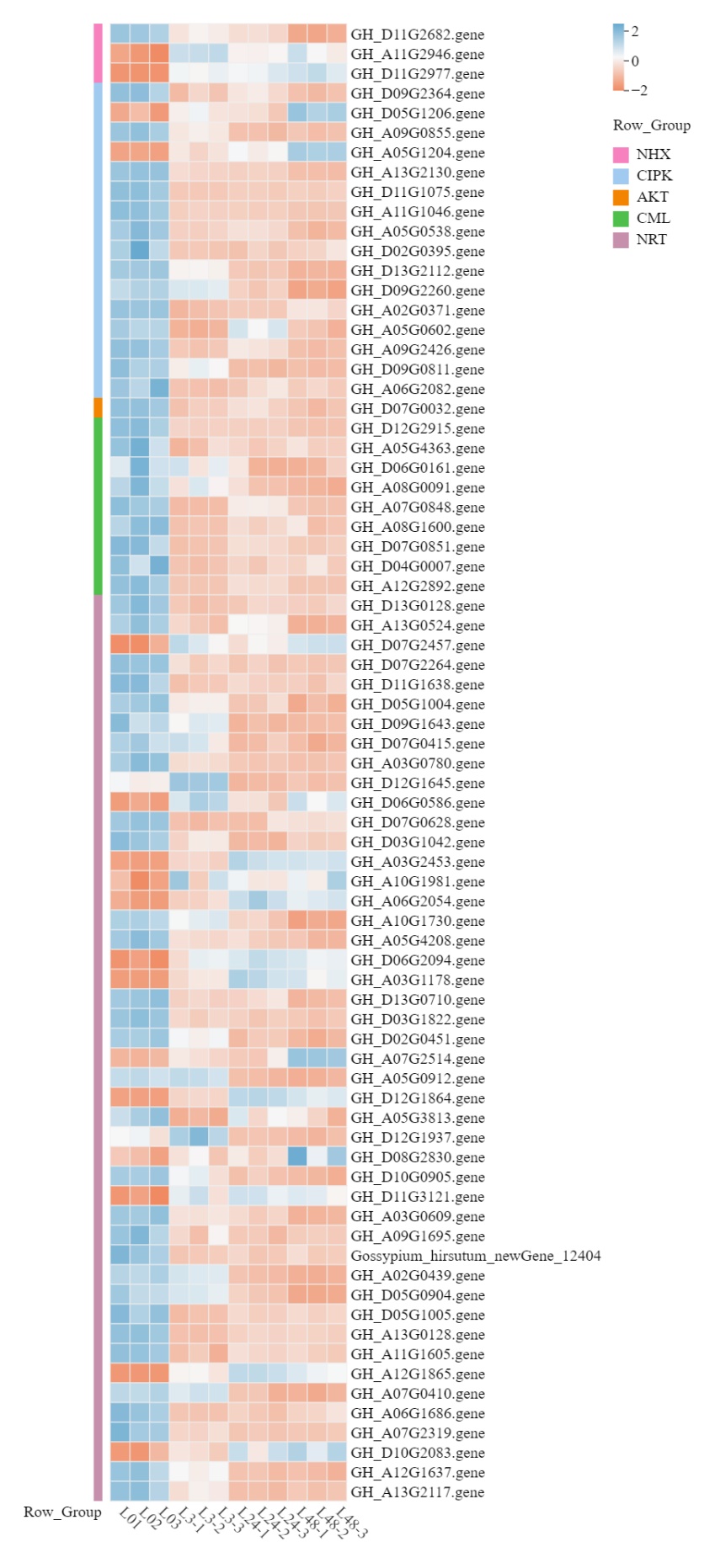
**

**Table S1** Evaluation statistics of sequencing data of samples in cotton

| Sample | Clean Reads | Clean Base(G) | Q30(%) | GC Content(%) |
| --- | --- | --- | --- | --- |
| L0-1 | 21141974 | 6.34 | 86.96 | 43.35 |
| L0-2 | 21342947 | 6.40 | 87.69 | 43.37 |
| L0-3 | 21238564 | 6.38 | 89.08 | 43.04 |
| L3-1 | 21353248 | 6.41 | 89.49 | 43.47 |
| L3-2 | 21315908 | 6.40 | 87.60 | 43.61 |
| L3-3 | 21098912 | 6.33 | 87.07 | 43.76 |
| L24-1 | 21270995 | 6.38 | 87.08 | 43.76 |
| L24-2 | 21327184 | 6.40 | 86.52 | 43.50 |
| L24-3 | 21285428 | 6.39 | 89.74 | 43.27 |
| L48-1 | 21457888 | 6.44 | 87.70 | 43.82 |
| L48-2 | 21208915 | 6.36 | 89.77 | 43.64 |
| L48-3 | 21110877 | 6.33 | 86.89 | 43.79 |

**Table S2** Comparison efficiency between sample and reference genome

| Sample | Reads mapped | Unique mapped |
| --- | --- | --- |
| L0-1 | 40920641(96.78%) | 39215566(92.74%) |
| L0-2 | 41380662(92.94%) | 39672365(92.94%) |
| L0-3 | 41262559(97.14%) | 39326560(92.58%) |
| L3-1 | 41595125(97.40%) | 39658479(92.86%) |
| L3-2 | 41423553(97.17%) | 39733301(93.20%) |
| L3-3 | 40919306(96.97%) | 39170075(92.82%) |
| L24-1 | 41337012(97.17%) | 39704202(93.33%) |
| L24-2 | 41434878(97.14%) | 39847708(93.42%) |
| L24-3 | 41568817(97.65%) | 39612684(93.05%) |
| L48-1 | 41780936(97.36%) | 40078130(93.39%) |
| L48-2 | 41414341(97.63%) | 39369610(92.81%) |
| L48-3 | 40992926(97.09%) | 39268299(93.00%) |

**Table S3** Verification of gene expression by qRT-PCR

| Gene name | Primer sequence |
| --- | --- |
| GH_D11G0140.gene-F | CGGTAACGTGAGCATCATCC |
| GH_D11G0140.gene-R | GGTCCTTGCGAATCTTCCAC |
| GH_D07G1800.gene-F | GTCCTGCGGTCATCTGTTTC |
| GH_D07G1800.gene-R | AGTAGGCCGGATCATCTTCG |
| GH_D02G0906.gene-F | TTGCGAATACGAGGATGTGC |
| GH_D02G0906.gene-R | GGGATCGAATGGTTTCTGCT |
| GH_A10G1631.gene-F | CTGGCATCAACTTTGGCCTT |
| GH_A10G1631.gene-R | GTCAGTGCTAGGGCTGATCT |
| GH_D08G1834.gene-F | GCTCGGACGGAGACAATAGA |
| GH_D08G1834.gene-R | GGCGTTCATCCAAATCGGAA |
| GH_A08G0887.gene-F | CAGAGCTACCTGGGCTTCTT |
| GH_A08G0887.gene-R | TGCTGGATTAGCAGTGGTGA |
| GH_A05G0504.gene-F | TGTGAACGTTGGAGGCAAAG |
| GH_A05G0504.gene-R | CCACATTCGGATCGTCATGG |
| GH_D07G2082.gene-F | GAGGTGGCTACCGAAACAAC |
| GH_D07G2082.gene-R | CTTTCCCTCCGTGTCCTCTT |
| GH_D12G1397.gene-F | AGGAGCATGTCATCAAGCCT |
| GH_D12G1397.gene-R | ATCTTACGTCCGGTGAGACC |
| GH_A03G1165.gene-F | CACCATGGGCGGAACATTAG |
| GH_A03G1165.gene-R | GTTTCCGGGAAGCTTCATCC |
| GH_D02G1331.gene-F | ATCTGACGGTGGAGGCTATG |
| GH_D02G1331.gene-R | CAGTAAATCGGCCAATGCCA |
| GH_A12G1381.gene-F | GTCCTCATCTCCACCCAACA |
| GH_A12G1381.gene-R | ATCTTACGTCCGGTGAGACC |
| GH_A03G1075.gene-F | AGATGCTTGAGGATGCTGGT |
| GH_A03G1075.gene-R | CGCCGCAGAACTTGTAAGAA |
| GH_D06G2043.gene-F | ACATGGAGTTGAGGCAGACA |
| GH_D06G2043.gene-R | GTGGCACGAATCTGAAACGA |
| GhActin-F | ATCCTCCGTCTTGACCTTG |
| GhActin-R | TGTCCGTCAGGCAACTCAT |
| GH_D10G0658.gene-F | GTGCAAAGGGAGGGTGATTG |
| GH_D10G0658.gene-R | AGCTTTAAGCCTGGGCAGTA |
